# Stress-induced mutagenesis: Stress diversity facilitates the persistence of mutator genes

**DOI:** 10.1371/journal.pcbi.1005609

**Published:** 2017-07-18

**Authors:** Marta Lukačišinová, Sebastian Novak, Tiago Paixão

**Affiliations:** Institute of Science and Technology Austria, Klosterneuburg, Austria; University of Texas at Austin, UNITED STATES

## Abstract

Mutator strains are expected to evolve when the availability and effect of beneficial mutations are high enough to counteract the disadvantage from deleterious mutations that will inevitably accumulate. As the population becomes more adapted to its environment, both availability and effect of beneficial mutations necessarily decrease and mutation rates are predicted to decrease. It has been shown that certain molecular mechanisms can lead to increased mutation rates when the organism finds itself in a stressful environment. While this may be a correlated response to other functions, it could also be an adaptive mechanism, raising mutation rates only when it is most advantageous. Here, we use a mathematical model to investigate the plausibility of the adaptive hypothesis. We show that such a mechanism can be mantained if the population is subjected to diverse stresses. By simulating various antibiotic treatment schemes, we find that combination treatments can reduce the effectiveness of second-order selection on stress-induced mutagenesis. We discuss the implications of our results to strategies of antibiotic therapy.

## Author summary

Many organisms display increased mutation or recombination rates when exposed to a stressful environment, which can increase the probability that the population acquires adaptations that allow it to avoid extinction. Because of this, it has been suggested that the increase in production rate of genetic variation is itself an adaptation. Here, we use a mathematical model to test this hypothesis. We find that this hypothesis is plausible when the environment is variable enough such that populations do not experience particular stresses too often. We provide an explicit expression for the critical time interval between exposures and discuss its implication for the evolution of resistance. Our results highlight how and when this form of evolvability can evolve by natural selection.

## Introduction

New mutations are the ultimate source of the variation that fuels adaptation. Accordingly, any mechanism that affects the rate at which new mutations are produced will impact a population’s ability to adapt. In a constant environment, well adapted populations are expected to evolve to lower mutation rates [1], while maladapted populations (*e*.*g*. under stress) frequently evolve mutator phenotypes [2]. These mutator phenotypes rise due to the short-term benefit of hitchhiking with beneficial mutations they induce, but suffer a penalty as the population approaches a fitness peak and the availability and/or effect of beneficial mutations decreases [3]. Consequently, elevated mutation rates persist most easily when selection pressure can be sustained despite adaptation. This is the case when populations experience fluctuating selection, which includes cells experiencing bursts of stresses during antibiotic treatment or cancer chemotherapy. Intuitively, if there exist mechanisms that increase mutation rates specifically during periods of stress, they could be selected since they provide the benefits of elevated mutations rates under stress while not incurring an additional mutation load in times when the population is well adapted.

There are multiple known mechanisms that result in elevated rates of general mutagenesis or an increase in the rate of specific genetic changes during stress. These mechanisms are often referred to as stress-induced mutagenesis (SIM). When encountering a range of environmental stresses, several species of bacteria activate SOS responses that—in addition to stimulating various repair mechanisms—activate error-prone DNA polymerases, which have been linked to a faster evolution of antibiotic resistance [4–6]. This activation of error-prone DNA polymerases in response to a wide range of evironmental stresses is a thoroughly studied SIM mechanism [4, 7]. The mutations are incorporated in proximity to DNA double-strand breaks under the condition that both the DNA damage activated SOS response and general stress response are active [8]. This mechanism has been linked to faster evolution of antibiotic resistance [5].

Several other such mechanisms have been identified. It has been shown that *Streptococcus pneumoniae* activates the expression of competence genes when treated with various antibiotics [9]. These genes allow the bacteria to take up DNA from the environment and incorporate it into its genome. Another example is the beneficial excision of a genomic region in the plant pathogen *Pseudomonas syringae* in response to the host’s immunity [10]. Similar mechanisms that link certain stresses to an increase of mutation rates have been found in *Drosophila melanogaster* [11] and yeast [12].

Several hypotheses may explain the prevalence of stress-induced mutagenesis. The first is a pleiotropic argument, presuming that SIM mechanisms are primarily due to first order selection for faster repair or nutritional gain (uptake of foreign DNA); then, the elevation of mutation rates is a side effect [13]. MacLean *et al*. [14] suggest an alternative hypothesis to explain the stress-linked induction of error-prone DNA polymerases: DNA polymerases that are linked to specific stress situations and that are used less often may be subject to weaker selection, and become error-prone by accumulation of slightly deleterious mutations. Another hypothesis, the second-order selection hypothesis, states that stress-induced mutagenesis has evolved due to its advantage of combining elevated mutation rates with those situations when they give most benefit [15, 16]. An allele that causes elevated mutation rates hitchhikes with the beneficial mutations it produces. By keeping mutation rates down at times of no stress, it reduces mutational load from excessive deleterious mutations compared to unconditionally increased mutation rates. There is no reason to think that only one of these hypotheses is correct; it is plausible that an interplay of these factors is responsible for the prevalence of SIM mechanisms in many organisms.

We explore the basic principle behind the second-order selection hypothesis of stress-induced mutagenesis: under which conditions and at what levels can a mechanism that increases mutation rates under stress be sustained in a population? What stress patterns and regimes promote it most? The relevance of these questions is imminent: stress-induced mutagenesis facilitates the adaptation of a population subjected to changing conditions. This is critical for cancer therapy or antibiotic treatment. Much effort goes into identifying strategies that keep the treatment effective for as long as possible, i.e., that impede the evolution of resistance [17–19]. If second-order selection is a key factor in the emergence and maintenance of SIM genes, however, different treatment regimes also affect the evolution of mutagenesis, and thus the evolvability towards resistance in the long term. It is therefore essential to understand to what extent different patterns of changing conditions cause second-order selection on stress-induced mutagenesis.

Previous studies have analyzed the evolution of mutator alleles by second-order selection in constant and variable environments. Some have focused on the evolution of mutation rates and the fate of constitutive mutator alleles [20, 21], which are predicted to be lost in constant and persist in fluctuating environments [22]. Closer to the system we study here, several models have investigated the evolution of fitness-dependent mutagenesis, where a decrease in fitness due to any deleterious mutation causes mutation rate to increase [16, 23, 24]. Interestingly, fitness-dependent mutator alleles are predicted to persist under a wide range of parameters in variable as well as constant environments [16]. To complement existing studies, we explore the persistence of a SIM allele which is strictly conditional on a stressful environment and cannot be triggered by a genetic change, since we focus on those SIM mechanisms which are dependent on environmental stress responses [7–9, 25].

We apply a mathematical model to investigate the plausibility of the second-order hypothesis for the evolution and maintenance of stress-induced mutagenesis (SIM). We show that populations subjected to diverse stresses can maintain SIM alleles as long as the period between exposure to these stresses is below a critical threshold. We provide analytical expressions for this critical threshold and show that there is an upper limit to the prevalence of such an allele, irrespective of the number of stressful environments a population is exposed to. Finally, in the context of the evolution of antibiotic resistance, we evaluate different scheduling alternatives of antibiotic therapies for their ability to prevent the maintenance of SIM alleles.

### A population genetic model for SIM alleles

**Fig 1:**
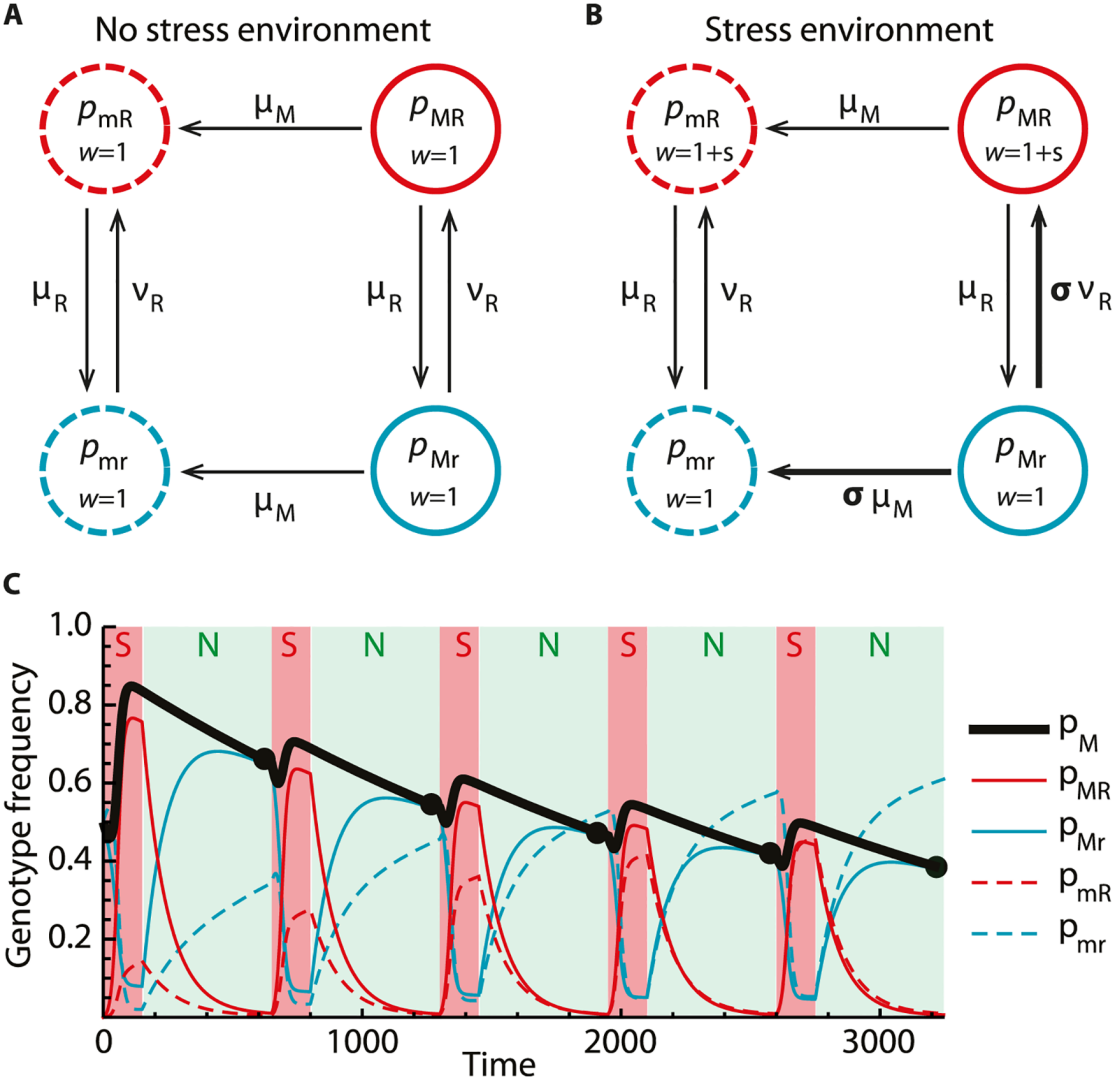
Schematic illustration of the SIM dynamics. (A) Under no stress, all genotypes have the same fitness *w* = 1 and transitions between the states are solely due to mutations. Resistance is lost and gained at rates *μ*_*R*_ and *ν*_*R*_, respectively. Furthermore, the SIM allele degrades at a rate *μ*_*M*_. (B) In the stress environment, individuals that are resistant to the stress gain a selective advantage *s* (fitness *w* = 1 + *s*). In addition, the genotype that is susceptible to the stress and carries the SIM allele (*p*_*Mr*_) increases its outgoing mutation rates by a factor *σ* > 1. (C) Periods of stress (S, red shading) and no stress (N, green shading) are alternated and the dynamics Eq (1) of genotype frequencies is simulated according to the schematic Fig 1A and 1B. During stress, resistant genotypes increase in frequency (red lines), and the SIM allele frequency *p*_*M*_ hitch-hikes (black line). If there is no stress, both resistance and SIM allele frequency levels decay. Over time, the SIM allele frequency thus fluctuates, possibly converging to stable oscillations. We sample the SIM allele frequency *p*_*M*_ at the end of each no-stress phase (black points), thus obtaining a discrete system in which the time between two successive measurements is given by the iteration of one cycle of stress and no stress.

We set up a model of a hypothetical stress-induced mutator (SIM) allele; its properties are based on the features of existing SIM mechanisms, yet focusing on the essence of a SIM mechanism independent of the molecular implementation. We are interested in exploring specifically the effectiveness of second-order selection in the evolution of a SIM allele. To do so, we need to isolate second-order selection from any direct benefit of the SIM system. Direct effects, for example faster DNA repair, are likely co-determinants of the persistence of SIM mechanisms in the wild, but such dynamics have also been extensively studied with existing evolutionary models, e.g. [26]. We therefore assume that the SIM allele does not confer any direct fitness cost or benefit, and consider a population of haploid individuals with two non-recombining loci. At the first locus, the SIM allele can be present or absent (alleles *M* or *m*, respectively), and the second locus carries alleles that may or may not grant resistance to a given stress (alleles *R* or *r*). The resulting four possible genotypes are displayed in Fig 1A and 1B.

In the absence of stress, we assume that transitions between the genotypes are only due to mutations, as indicated by the arrows in Fig 1; thus in particular, we assume that there is no cost to being resistant. Individuals may lose or gain resistance at rates *μ*_*R*_ and *ν*_*R*_, respectively. The SIM allele *M* may lose its function at rate *μ*_*M*_; since we are interested in conditions for the ultimate loss of the SIM allele, we neglect back-mutation from *m* to *M*.

In the stress environment, genotypes containing the resistance allele *R* have increased fitness *w* = 1 + *s* relative to susceptible genotypes. Furthermore, the *Mr* genotype increases all outgoing mutation rates by a factor *σ* > 1 due to stress-induced mutagenesis, see Fig 1B. Key assumptions behind this modelling approach are: First, stress does not activate the SIM allele in resistant individuals. This is reasonable if, for example, the stressor is effective inside the cell but the resistant mutation makes the cell membrane impermeable to it. Second, the only cost of an active SIM allele is that it increases the rate of its own loss. This at best partially represents the detrimental effects of elevated mutation rates not considered in this model. However, artificially creating an idealised situation for the SIM allele allows us to keep the model tractable (but see S3 Appendix for a treatment of lethal mutations).

We cast the schematic dynamics of Fig 1 into two sets of differential equations for the variables *p* = {*p*_*mr*_, *p*_*Mr*_, *p*_*mR*_, *p*_*MR*_}. Using the classical mutation-selection dynamics of population genetics, they take the form [27]

$$\begin{array}{c} \dot{p}=p(w-\bar{w})+M.p, \end{array}$$ (1)

where *w* is the vector containing the marginal fitnesses of the genotypes, $\bar{w}$ is the mean fitness of the population, and M is a matrix encoding the mutation scheme (see Methods for the explicit set of equations). In order to make analytical progress, we make a number of simplifying assumptions. First, we assume that selection under stress is strong compared to mutation. This is justified in treatment-like scenarios we consider here. Second, we assume that the mutation rate leading to a loss of a resistance mechanism is larger than the mutation rate leading to its gain. This is plausible, since by random genetic modifications it is more likely to disable a functional mechanism than to create one. Hence, it seems reasonable to assume the following hierarchy among the parameters:

$$\begin{array}{c} s\gg \left(\mu_{M},\mu_{R}\right)\gg \nu_{R}. \end{array}$$ (2)

Given this hierarchy, it can be shown that the SIM allele is not maintained in any of the two environments separately. Switching between the stress and no-stress environments, however, gives rise to non-trivial dynamics. During a stress phase, the SIM allele may increase in frequency along with the resistance mutations it produces. As resistance levels in the population rise, this effect weakens and the SIM allele frequency falls off because of mutations degrading the SIM mechanism. Periods of no stress allow resistance levels to decline, such that the SIM allele becomes effective again in the next stress phase.

## Results

### SIM alleles are maintained at higher frequencies under diverse stresses

In order to obtain analytical insight into the dynamics of SIM alleles we first consider two extreme scenarios: the recurrent (*R*) and non-recurrent (*NR*) stress scenarios. In both scenarios, an infinitely large population is subjected to an environment that alternates between periods of stress (for *τ*_*S*_ time units) and of no stress (for *τ*_*NS*_ time units). In the recurrent scenario, the stress periods are assumed to be all the same (i.e. resistance acquired in the previous stress period carries over to the next stress period). In the non-recurrent stress scenario we assume that each new stress period is different such that resistance acquired in previous stress periods is not beneficial in any subsequent stress period.

In both regimes, the genotype frequencies evolve as described by the dynamical system Eq (1) and according to the schematics in Fig 1. Iterating this procedure leads to oscillations in the SIM allele frequency *p*_*M*_ = *p*_*Mr*_ + *p*_*MR*_ as depicted in Fig 1C. We measure genotype frequencies at discrete time points directly before the onset of each stress period (bold points in Fig 1C). The long-term equilibria of this time series thus describe the long-term prevalence of the SIM allele, which we denote by ${\hat{p}}_{M}$. Since our model assumes an effectively infinite population, the SIM allele cannot be lost within one cycle. Nevertheless, it is possible that the SIM allele frequency asymptotically declines to zero as the cycles are iterated (i.e., that ${\hat{p}}_{M}=0$).

We assume that during stress selection is strong relative to mutation, and that the effect of the SIM allele is large. As a consequence, the stress dynamics has two phases; during the rapid first phase, genotype frequencies are almost exclusively due to selection (*s*) and those mutation rates that are amplified by the SIM allele (*σμ*_*M*_ and *σν*_*R*_). At the end of the first phase, almost all individuals have acquired resistance to the stress. In the second, slower, phase, resistance levels remain high and the SIM allele slowly degrades due to mutation (*μ*_*M*_). We further assume that stresses are of short duration, so that we may ignore this second phase. Mathematically, we replace *s* ↦ *αs* and *σ* ↦ *ασ*, rescale time by *dt* ↦ *dt*/*α*, divide by *α*, and let *α* → ∞ (see the Methods section and S1 Appendix for details).

We aim to calculate the SIM allele frequencies *p*_*M*_ = *p*_*Mr*_ + *p*_*MR*_ before the onset of each stress period, *i*.*e*., at the end of each cycle of stress followed by no stress. Under stress, the relative proportions of the *mR* and *MR* genotypes are maintained except for an excess of *MR* genotypes being generated by amplified mutation from the *Mr* genotypes. This excess is *ν*_*R*_/(*s*/*σ* + *μ*_*M*_+*ν*_*R*_). In the absence of stress, resistance levels relax to *p*_*R*_(*τ*_*NS*_), which approaches mutation balance (*ν*_*R*_/(*μ*_*R*_ + *ν*_*R*_)) for long periods without stress (*τ*_*NS*_ → ∞). At the same time, the frequency of the SIM allele decays exponentially due to mutations from its initial value *p*_*M*_(0) to *p*_*M*_(0) *exp*(−*μ*_*M*_ *τ*_*S*_). Heuristically, the SIM allele frequency $p_{M}^{\prime }$ before the next stress is thus obtained from the SIM allele frequency *p*_*M*_ before the current stress as

$$\begin{array}{c} p_{M}^{\prime }=p_{M}\;e^{-\mu_{M}\;\tau_{NS}}\;\frac{1+\lambda }{1+p_{M}\;\lambda }, \end{array}$$ (3)

where λ = (1 − *p*_*R*_(*τ*_*NS*_))/*p*_*R*_(*τ*_*NS*_) *ν*_*R*_/((*s*/*σ* + *μ*_*M*_ + *ν*_*R*_). This intuitive derivation of the dynamics is made precise in S1 Appendix, where we also calculate *p*_*R*_(*τ*_*NS*_) for the recurrent stress (*R*) scenario. In the non-recurrent (*NR*), we have *p*_*R*_(*τ*_*NS*_) = *ν*_*R*_/(*μ*_*R*_ + *ν*_*R*_), since resistance levels to yet unknown stresses can be assumed to be at mutation balance. Solving Eq (3) for equilibria yields the long-term prevalences of the SIM allele in the (*R*) and (*NR*) scenarios as

$$\begin{array}{cc} {\hat{p}}_{M}^{\left(R\right)} & =\text{max}\{0,\;e^{-\mu_{M}\tau }-\Gamma \left(1-e^{-\mu_{M}\tau }\right)\cdot \\ & \cdot \left(1+\frac{\mu_{R}+\nu_{R}}{\nu_{R}}{\left(e^{\left(\mu_{R}+\nu_{R}\right)\tau }-1\right)}^{-1}\right)\}, \end{array}$$ (4a)

$$\begin{array}{cc} {\hat{p}}_{M}^{\left(NR\right)} & =\max\{0\;,\;e^{-\mu_{M}\tau }-\Gamma (1-e^{-\mu_{M}\tau })\} \end{array}$$ (4b)

(see S1 Appendix), where *τ* = *τ*_*S*_ + *τ*_*NS*_ is the length of one cycle of stress and no-stress, and

$$\begin{array}{c} \Gamma =\frac{s/\sigma +\mu_{M}+\nu_{R}}{\mu_{R}}. \end{array}$$ (5)

In particular, we thus see that the stress intensity *s* and the strength of the SIM allele *σ* enter the long-term SIM allele prevalences only via their ratio *s*/*σ*.

**Fig 2:**
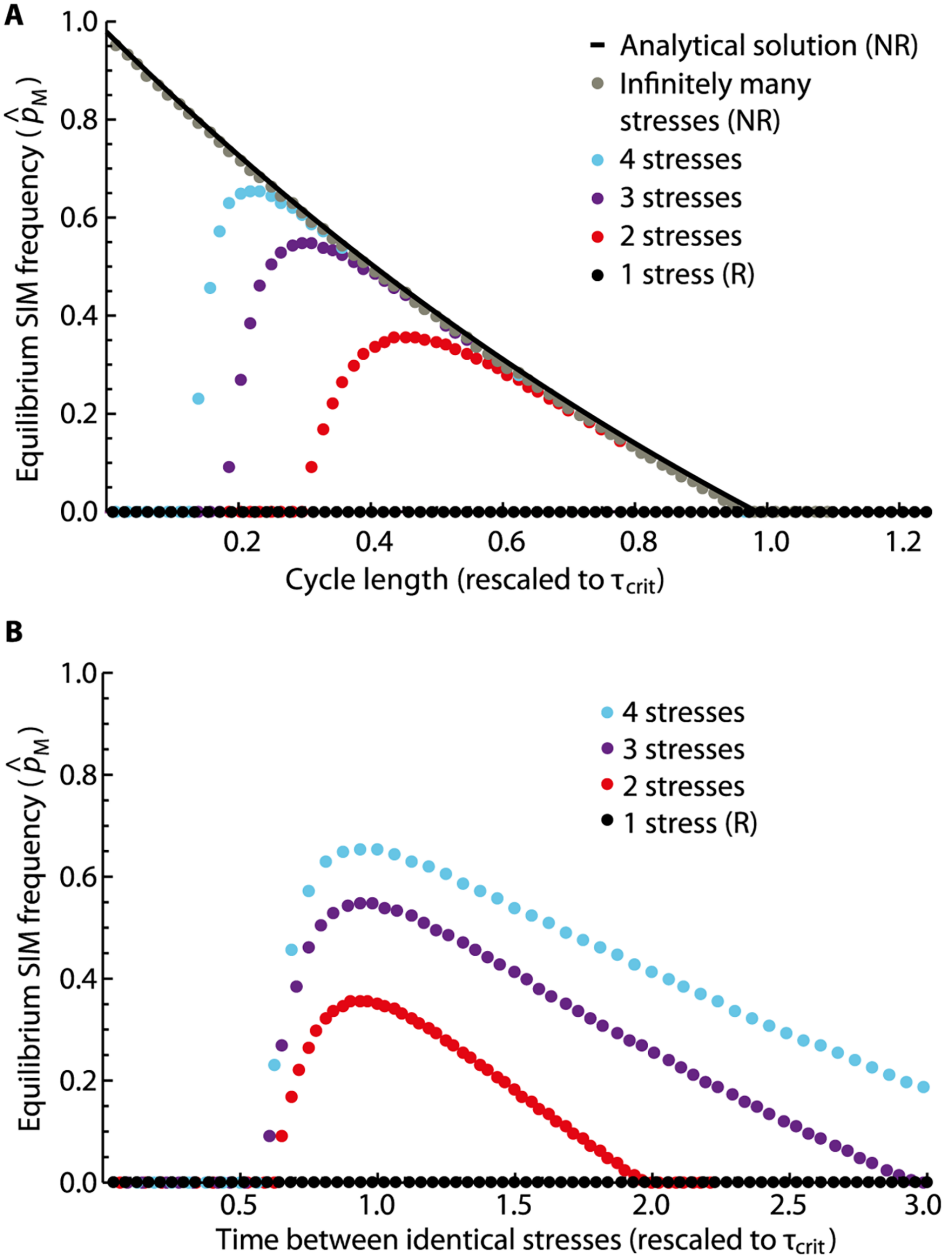
SIM prevalences increase with stress diversity. A representative parameter set (*σ* = 100, *s* = 1, *μ*_*M*_ = 10^−3^, *μ*_*R*_ = 10^−2^, and *ν*_*R*_ = 10^−4^) was simulated for a range of values of *τ*/*τ*_*c*_ = (*τ*_*S*_ + *τ*_*NS*_)/*τ*_*c*_. The solid black lines represent the analytical predictions from Eq (4) for the (*R*) and (*NR*) regimes. For the numerical simulations, we chose *τ*_*S*_ = 10 and varied *τ*_*NS*_ accordingly. The simulation results of the (*R*) and (*NR*) regimes (black and grey points) fit their corresponding predictions well. The red, purple, and blue points represent simulation results for two, three, and four different stresses occurring cyclically. Increasing the number of stresses increases the SIM prevalences up to a maximum given by the prediction for the (*NR*) regime. (A) The critical cycle length *τ*_*c*_ determines the maximal stress re-occurrence time that allows for the maintenance of the SIM allele. (B) The minimal stress re-occurrence time that permits positive long-term SIM prevalences is determined by the time between identical stresses. This time is 2*τ* (3*τ*, 4*τ*) for two (three, four) stresses.

To test our analytical predictions, we explicitly simulate the dynamics (Eq (1)) of a population experiencing stress and no-stress phases according to the schematics in Fig 1 without the simplifications that lead to the above formulae. Fig 2A shows the long-term SIM prevalences as functions of the cycle length *τ* for a representative choice of the remaining parameters. For both the (*R*) and (*NR*) regimes, the simulated values (points) align well with the above formulae (solid lines). In the non-recurrent regime, the SIM allele is maintained in the population as long as stresses occur frequently enough; more precisely, there is a critical cycle length *τ*_*c*_ such that the SIM allele is not maintained for cycle lengths exceeding *τ*_*c*_,

$$\begin{array}{c} {\hat{p}}_{M}^{\left(NR\right)}=0\;\;\text{if}\;\;\tau >\tau_{c}=\frac{1}{\mu_{M}}\log(1+\frac{1}{\Gamma }). \end{array}$$ (6)

Furthermore, in this regime there is a strictly monotone dependence between the steady state SIM allele frequency and the frequency of stress occurrence; in particular, the SIM allele becomes fixed in the population in the limit of infinitely rapid stress occurrence (i.e., ${\hat{p}}_{M}^{\left(NR\right)}\to 1$ for *τ* → 0).

In the recurrent regime, the dependence of the equilibrium SIM levels ${\hat{p}}_{M}^{\left(R\right)}$ on the cycle length *τ* is less simple. If the rate of gaining resistance without the SIM allele is sufficiently low (i.e., *ν*_*R*_ ≪ 1, in particular *ν*_*R*_ ≪ *μ*_*R*_), the SIM allele is not maintained in the population for any choice of *τ* (Fig 2A, see also S1 Appendix). Note that in general there are conditions that do lead to the maintenance of SIM alleles in the recurrent regime. Such cases, however, are not in concordance with our basic ranking of parameters, inequality Eq (2) (see S1 Appendix). Furthermore, we show in S1 Appendix that the non-recurrent regime generally maintains a higher SIM prevalence than the recurrent regime, i.e., ${\hat{p}}_{M}^{\left(NR\right)}\geq {\hat{p}}_{M}^{\left(R\right)}$ for any choice of parameters.

We can extend our basic model to include additional biologically relevant factors, such as cost of resistance or the presence of lethal mutations (see S2 Appendix). These factors change the long-term SIM prevalences in intuitive ways, yet leave our qualitative statements unchanged. For example, maintaining a resistance mechanism in the absence of stress may incur a fitness cost. Consequently, resistance levels decrease faster in the no-stress environment if resistance is costly, which increases the benefit of increased mutation rates to acquire resistance under stress. Accordingly, including a cost of resistance to our model increases the long-term SIM prevalences (see Fig B in S2 Appendix).

We observe the opposite effect if we consider a mutational load due to lethal mutations. The greater risk for mutator phenotypes to acquire deleterious mutations can be expected to cause indirect selection pressure against the SIM allele. Describing a gradual accumulation of deleterious mutations requires the consideration of multiple fitness classes, which is infeasible in our approach. Instead, we show in S3 Appendix (see Fig D in S3 Appendix) that lethal mutations translate into selection against the SIM allele and thus reduce long-term SIM prevalences.

### SIM prevalence increases with number of sequentially applied stresses

We explore the prevalence of the SIM allele when subjected to a finite number of stresses. To this end, we simulate the full system as explained earlier for the (*R*) and (*NR*) regimes, but for a finite number *χ* of challenges. This is done by taking into account a separate resistance locus for every challenge. Each of these extra resistance loci is neutral during non-cognate environmental challenges. During this time period, their frequency changes only by mutational degradation or if they are associated to the resistance allele that is under selection. As in the (*NR*) regime, we assume no cross-resistance and there is complete linkage between all loci. Hence, there are 2^*χ*+1^ different genotypes to consider. The stresses are applied in a deterministically cycling manner. Each stress period is of constant length *τ*_*S*_, and is followed by a no-stress period of length *τ*_*NS*_.

The results interpolate between the (*R*) and (*NR*) regimes, where every increase in the number of stresses, *χ*, also increases the SIM allele equilibrium frequency and the parameter regime where it is maintained (Fig 2). In particular, the simulations suggest a simple classification of the possible dynamical regimes, based on the length of the stress periods (*τ* = *τ*_*S*_ + *τ*_*NS*_).

First, for small values of *τ*, we observe the loss of the SIM allele. The upper bound of this region is inversely proportional to the time it takes for the same stress to recur. Keeping the cycle length *τ* constant and increasing the number of challenges *χ* also increases this time and therefore allows for the maintenance of the SIM allele for smaller values of *τ*. The scaling with the time between two stresses of the same type can be seen clearly in Fig 2B. We may deduce that a too frequent occurrence of the same stress is not beneficial for the SIM allele. This is not surprising; the SIM allele has no fitness advantage on its own and therefore can only rise in frequency if the relevant resistance levels in the population are low. When stresses re-occur frequently, resistance levels are kept high, preventing the SIM allele from hitchhiking.

Second, if the number of different stresses is high enough, a SIM allele can be kept for intermediate frequencies of stress occurrence. The size of this region expands with increasing stress diversity up to the level of the (*NR*) regime of infinite stress diversity. The maximum allele frequency that can be kept also increases with increasing stress diversity, geometrically approaching the analytically determined value of the (*NR*) case.

Third, if stresses occur too infrequently, the SIM allele is lost. The critical time between two consecutive stresses, above which the SIM allele is lost for any number of stresses *χ*, was calculated analytically as *τ*_*c*_, see Eq (6).

With *χ* different stresses, each particular stress occurs every *χτ* time units. Assuming that resistance alleles to different stresses do not interact, we thus may replace *p*_*R*_(*τ*_*NS*_) by *p*_*R*_(*χτ*_*NS*_) in the heuristic derivation of the recursion Eq (3) to obtain an approximation to the dynamics of SIM allele frequencies with *χ* different stresses. In our actual model, however, resistance mutations to different stresses do not evolve independently since they are linked to the genetic backgrounds they appear on and cross resistance against multiple stresses is possible. The approximation thus captures the qualitative behaviour of the long-term SIM allele frequencies for multiple stresses, yet overestimates the numerical results for the parameters used in our simulations, (see S3 Appendix, in particular Fig D).

**Fig 3:**
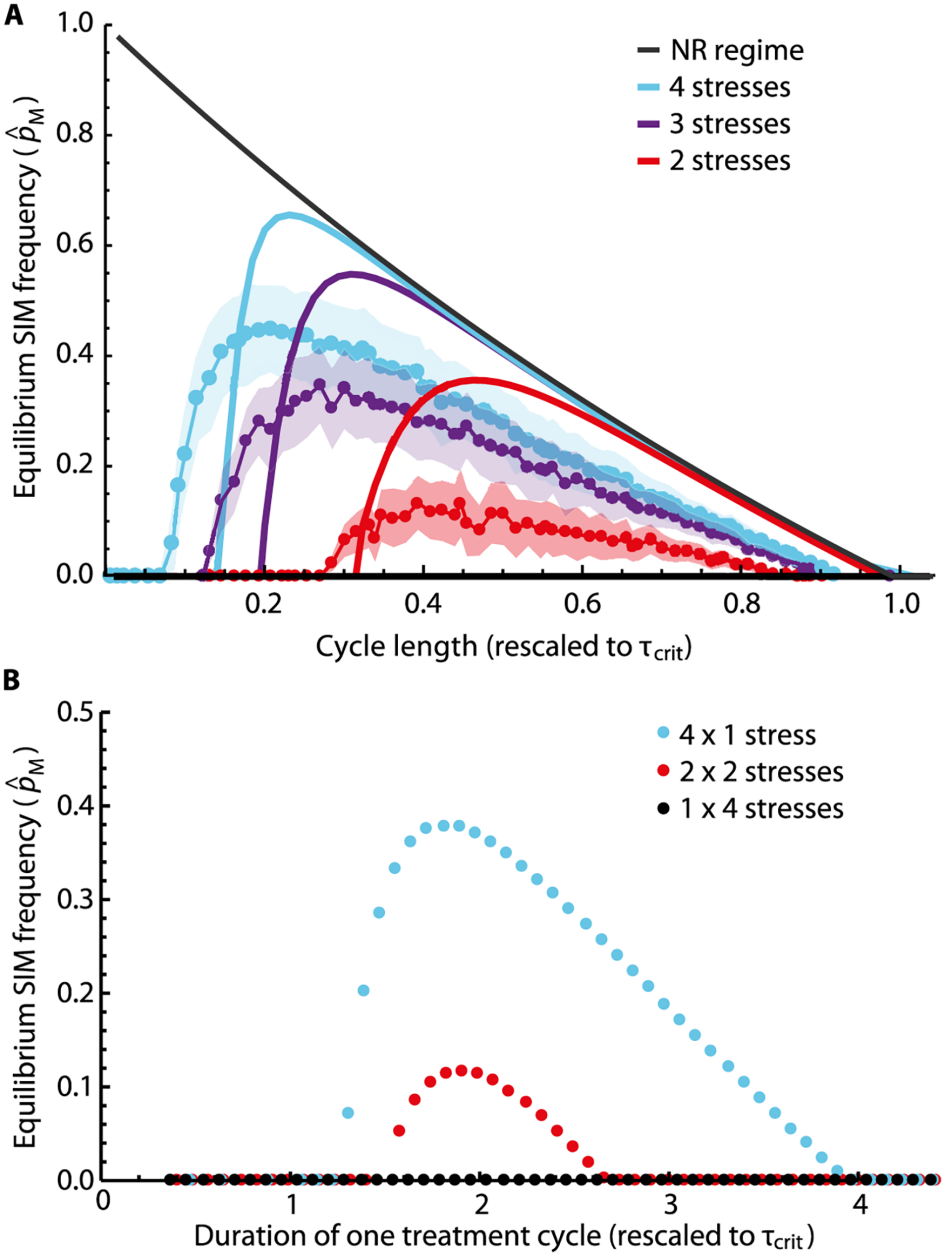
The effect of stochasticity and grouping of stresses on SIM prevalences. (A) The solid lines represent the same data as Fig 2. In addition, we randomized the simulation by choosing the next stress randomly from the set of available stresses (instead of a deterministic periodic stress cycle). 10,000 iterations of randomly chosen stress and no stress were performed, and the SIM prevalences over the last 1,000 were calculated (red, purple, and blue points for two, three, and four different stresses). The shaded areas indicate the standard deviations in the samples and there is a clear increase in SIM prevalence levels for increasing numbers of different stresses. However, the mean SIM prevalences are significantly lower than corresponding long-term SIM prevalences from deterministic simulations. (B) To mimic treatment regimes, we simulated the simultaneous versus sequential occurrence of four different stresses. We assumed that resistance to each stress confers a selective advantage *s*. If multiple stresses occur simultaneously, their effects add up such that, for instance, being resistant against two simultaneously occurring stresses provides an advantage of 2*s*. If all four stresses occur simultaneously (combined treatment) every *τ* time units, the SIM allele is not maintained for our chosen set of parameters (black points). In contrast, if the four stresses are applied in sequence (sequential treatment) with *τ*/4 time units between consecutive stresses—such that one cycle through all stresses takes *τ* time units—the SIM allele is maintained at considerable frequency for a wide range of values of *τ* (blue points). Grouping the four stresses in two pairs and alternating those at half the previous rate (*τ*/2; each pair of stresses re-occurs every *τ* time units) leads to intermediate SIM allele maintenance levels (red points). (Parameters: *σ* = 100, *s* = 0.5, *μ*_*M*_ = 0.001, *μ*_*R*_ = 0.005, *ν*_*R*_ = 0.0001.)

To relax our assumption of stresses occurring in a strict cycle, we randomize our model by choosing one out of the *χ* stresses at each iteration of the simulation. Qualitatively, this leaves the picture unchanged, see Fig 3A: The SIM prevalence levels ${\hat{p}}_{M}$ and the interval of stress occurrence times *τ* that maintain the SIM allele both increase with increasing stress diversity, though not as readily as in the deterministic case. However, a shift can be seen in which values of *τ* make maintenance of SIM possible, leading to a small interval of cycle lengths *τ* when randomization enables SIM allele maintanence. This happens because the effective time interval between two identical stresses is now a random variable, and there is some probability that the same stress is seen sooner than in the deterministic regime. This means that a cycle length, that in the deterministic regime is not conducive to the maintenance of the SIM allele, can now sustain it because there is some probability that the same stress is seen at an interval that does support it. One important point is that the minimum time interval between two identical stresses is the time of cycle. This means that the distribution of time intervals is right-skewed, which explains why the “shift” seen on the simulation curves is to the left (the simulations “sample” times to the right).

### Combination treatments prevent the rise of SIM alleles

For practical questions in antibiotic therapy, it is of interest to investigate treatment scenarios in which a set of pharmaceuticals is administered simultaneously or separately over a given period of time (combined versus sequential treatment [17, 28, 29]). To this end, we simulate and compare four stresses either occurring simultaneously, being grouped in two pairs, or being applied separately. We assume that the stresses do not allow for cross-resistance mutations (i.e., single mutations that provide resistance to multiple stresses), that their effects on fitness in genotypes with multiple resistance mutations are additive, and that one cycle through all stresses or stress combinations takes *τ* time units in each case. The results of our simulations are depicted in Fig 3B: while applying all stresses at once does not maintain the SIM allele for our choice of parameters (Fig 3B, black), the SIM allele prevalence increases if stresses occur more frequently, yet in a less clustered fashion (Fig 3B, red and blue). We have also measured the levels of multi-resistance in these scenarios (S4 Appendix). Interestingly, we find that for short treatment cycles (in which the SIM allele is not expected to be maintained) a mixed strategy in which different sets of multiple stresses are applied sequentially seems to perform best at avoiding multi-resistance, even if at the cost of a higher prevalence of single resistant strains (see Table A in S4 Appendix).

## Discussion

Our study investigates the fate of a hypothetical stress-induced mutagenesis (SIM) mechanism under various schemes of environmental fluctuation. We assume that stress-induced mutagenesis is brought about by an active mechanism that increases mutation rates as a response to stress, modeled by a modifier allele for stress-induced mutagenesis that is much easier lost than gained. As a consequence, it decays over time unless maintained by recurrent second-order selection due to changes in the environment. This is what would be expected under the adaptive hypothesis which we are testing. Our results indicate that there are plausible regimes under which the SIM allele could be kept purely by second-order selection under the adaptive hypothesis. What is needed is that the basic hierarchy of parameters outlined in Eq (2) is met. This is reasonable if one considers relatively strong stress episodes and a resistance mechanism such as an antibiotic degrading beta-lactamase enzyme, which is difficult to acquire, but easier to degrade by mutation [30]. Furthermore, a regime of environmental fluctuations is needed such that resistance levels are not kept very high between repeated strikes of the same stress, which can be aided in natural populations by fitness costs associated with resistance mutations [31](see also S2 Appendix). Also, stresses in which SIM helps bring about a beneficial mutation need to occur frequently enough to prevent the degradation of SIM. Finally, stress diversity greatly facilitates the maintenance of SIM by requiring resistance mutations that are new or less prevalent in the population. Considering that bacteria in a human body can often experience starvation, acid stress, inflammation, or treatment induced antibiotic stress, these conditions are also plausible [32, 33]. Although the maintanance of SIM due to second order selection is plausible, our model tends to underestimate SIM frequencies observed in natural populations which are close to 100% [4]. Direct benefits of SIM mechanisms [34] or a high cost of resistance mutations are common phenomena which are expected to increase the frequency of SIM alleles and could explain the higher frequency found in nature.

Under our assumptions, environmental fluctuations are essential for the SIM allele to be maintained in the population: in the absence of environmental challenges (stresses), the SIM allele is lost due to the neutral accumulation of loss-of-function mutations. Repeatedly occurring stresses, however, give rise to second-order selection on the SIM allele. Under reasonable assumptions on the model parameters, c.f. Eq (2), we show that simple fluctuations caused by a repetitive stress generally fail to maintain the SIM allele. As the stress diversity—i.e., the number of different stresses available—increases, the SIM allele may be maintained at increasingly high levels (see Fig 2). In the limit of infinite stress diversity, the SIM allele is maintained for any frequency of stress occurrence above a given threshold, which we characterized analytically by *τ*_*c*_.

Interestingly, when a fixed number of stresses are applied in a random order, the prevalence levels of the SIM allele generally decrease, and the parameter region conducive to maintenance shifts: maintanence can happen at shorter time intervals, and *τ*_*c*_ is apparently reduced (Fig 3A). This is because in this scenario, the time between two stresses of the same kind is now stochastic: there is a probability distribution for the time a particular stress is re-applied. This effectively “smoothes” the deterministic expectation for the steady state frequency. This leads to the “shift” of the simulation curves seen on Fig 3.

It should be noted that we model the dynamics of an infinite population which prevents the examination of the stochastic effects introduced by genetic drift. In our model the SIM allele can never truly fix or be lost from the population. The first point is not very consequential, since it is natural to assume that deleterious mutation will always act to degrade the SIM mechanism and lower its frequency from fixation. However, the second point may be more important since mutations that reintroduce the SIM mechanism after it has been lost may be rare. However, our results can still provide some insights: if the frequency of the SIM allele drops below 1/*N*, where *N* is the population size, one can say that it is effectively lost. Furthermore, it is not clear if the rate of back-mutations in nature is effectively zero. If indeed there is some probability of reintroducing the SIM mechanism then our deterministic results provide an expectation for its long-term frequency.

Our results focus on how the maintenance of a SIM allele depends on the frequency and diversity of stresses. We find that in the case of cycling a finite number of stresses, the SIM allele can only be maintained at intermediate stress frequencies. Irrespective of the number of available stresses, a lower bound for the stress frequency can be determined analytically as 1/*τ*_*c*_. For the upper bound, we find that the time between two stresses of the same kind is crucial (Fig 2B). This could inform the choice of therapeutic strategies by identifying treatment schedules that exert extensive selection pressure to keep a SIM allele and possibly strengthen its effect.

To date, various temporal treatment strategies have been investigated to counter the current antibiotic resistance crisis [35–37]. To prevent the emergence of resistant strains, one approach is to inhibit known resistance mechanisms directly [38]. Another is to use combinations of existing drugs in treatment regimes that are rationally designed to suppress resistance levels [39, 40]. However, to keep drugs effective in the long term, it is desirable to develop strategies that not only decrease resistance levels, but also restrict evolvability. To this end, there have been efforts to directly inhibit SIM mechanisms [5, 41]. Our study complements this approach by assessing how temporal treatment schemes prevent second-order selection on a SIM mechanism. We find that an increasing diversity of stresses encountered increases long-term SIM frequencies (see Figs 2 and 3B). This suggests a trade-off between controlling resistance and controlling evolvability when designing multi-drug therapies: in most proposed schemes, one tries to prevent the evolution of resistance by diversifying the stresses (antibiotics) [35–37]. However, our findings suggest that this is precisely the scenario in which SIM alleles are more likely to persist and hence promote the evolvability of the population. Experimental work is needed to further characterize this trade-off and assess its relevance in a clinical setting. Currently, it is known that SIM mechanisms are common in bacteria, vary greatly in their potency [4] and can be lost due to a variety of mutations [25]. Selection pressures we describe here could therefore favor those strains that have a significantly higher mutation rate in stress also in the clinic. To confirm this relevance, studies measuring temporal dynamics of SIM alleles in a clinical setting are needed. Also, long-term microbial evolution experiments in a more controlled setting that would follow the prevalence of a synthetic or natural SIM allele over time under different treatment schemes are plausible. Our results may inform such experiments to confirm the suggested trade-off between the evolution and evolvability of resistance.

It has been proposed that the simultaneous application of drugs that exhibit no cross-resistance may be more effective against resistant strains than their sequential application [17, 28, 29], but also the opposite [42]. In our model, the same applies to reducing positive second-order selection on SIM alleles. Exploring this finding further may provide a resolution of the trade-off between fighting resistance and evolvability, at least for those drug combinations that allow for simultaneous application despite common toxicity or dosage problems.

## Methods

### Differential equation model

Casting the schematic dynamics of Fig 1 into differential equations of the form Eq (1) yields

$$\begin{array}{c} {\dot{p}}_{mr}=\mu_{M}\;p_{Mr}+\mu_{R}\;p_{mR}-\nu_{R}\;p_{mr}, \end{array}$$ (7a)

$$\begin{array}{c} {\dot{p}}_{Mr}=\mu_{R}\;p_{MR}-\left(\mu_{M}+\nu_{R}\right)\;p_{Mr}, \end{array}$$ (7b)

$$\begin{array}{c} {\dot{p}}_{mR}=\nu_{R}\;p_{mr}+\mu_{M}\;p_{MR}-\mu_{R}\;p_{mR}, \end{array}$$ (7c)

$$\begin{array}{c} {\dot{p}}_{MR}=\nu_{R}\;p_{Mr}-\left(\mu_{M}+\mu_{R}\right)\;p_{MR}, \end{array}$$ (7d)

for the no-stress environment. This system of ordinary differential equations can be solved explicitly. Given an initial SIM allele frequency $p_{M}^{*}$, we find that $G\left(p_{M}^{*}\right)=p_{M}^{*}\exp[-\mu_{P}\;\tau_{NS}]$ is the SIM allele frequency after *τ*_*NS*_ time units of no stress, see S1 Appendix. For the stress environment, we have

$$\begin{array}{c} {\dot{p}}_{mr}=-s\;p_{mr}(p_{mR}+p_{MR})+\sigma \mu_{M}\;p_{Mr}-\nu_{R}\;p_{mr}+\mu_{R}\;p_{mR}, \end{array}$$ (8a)

$$\begin{array}{c} {\dot{p}}_{Mr}=-s\;p_{Mr}(p_{mR}+p_{MR})-\sigma (\mu_{M}+\nu_{R})p_{Mr}+\mu_{R}\;p_{MR}, \end{array}$$ (8b)

$$\begin{array}{c} {\dot{p}}_{mR}=s\;p_{mR}(1-p_{mR}-p_{MR})+\nu_{R}\;p_{mr}-\mu_{R}\;p_{mR}+\mu_{M}\;p_{MR}, \end{array}$$ (8c)

$$\begin{array}{c} {\dot{p}}_{MR}=s\;p_{MR}(1-p_{mR}-p_{MR})+\sigma \nu_{R}\;p_{Mr}-(\mu_{R}+\mu_{M})p_{MR}. \end{array}$$ (8d)

Assuming that stress is strong and of short duration, and that the SIM allele has a large effect, we may replace *s* ↦ *αs*, *σ* ↦ *ασ*, and rescale time *dt* ↦ *dt*/*α*. Dividing by *α* and letting *α* → ∞, Eq (8) simplifies (see S1 Appendix) and permits an approximation for the SIM allele frequency after a short period of stress. We write $p_{M}^{*}=F\left(p_{M}\right)$ for the SIM allele frequency after stress; the mapping F is derived in S1 Appendix and depends on whether stress is recurrent or non-recurrent (the (*R*) and (*NR*) regimes). Measuring genotype frequencies directly before each stress, we thus obtain a recursion for the SIM allele frequency *p*_*M*_ as

$$\begin{array}{c} p_{M}^{\prime }=(G\circ F)\left(p_{M}\right), \end{array}$$

which can be written as Eq (3). Solving this recursion for $p_{M}^{\prime }=p_{M}$ leads to the long-term SIM allele prevalences in Eq (4).

### Simulations

Our numerical simulations were implemented using the software *Mathematica*. For a single recurrent stress (the (*R*) regime), we alternate periods of stress (dynamics Eq (8)) for *τ*_*S*_ time units with periods of no stress (dynamics Eq (7)) for *τ*_*NS*_ time units. Genotype frequencies are recorded before each stress period, and the procedure is stopped after 10^4^ iterations or once the genotype values reach an equilibrium. To simulate the (*NR*) regime, we proceed likewise but replace the genotype frequencies {*p*_*mr*_, *p*_*Mr*_, *p*_*mR*_, *p*_*MR*_} before every stress by

$$\begin{array}{c} \{(1-\varepsilon )(p_{mr}+p_{mR}),(1-\varepsilon )(p_{Mr}+p_{MR}),\\ \varepsilon (p_{mr}+p_{mR}),\varepsilon (p_{Mr}+p_{MR})\} \end{array}$$

before every new stress, where *ε* = *ν*_*R*_/(*μ*_*R*_ + *ν*_*R*_). Since the particular kind of stress has never occurred before, the probability of being resistant to it is given by the balance *ε* between the rates of gaining and losing resistance due to mutation.

With *χ* > 1 different stresses, there are 2^*χ*+1^ different genotypes. We consider only single point mutations; the SIM allele is lost at rate *μ*_*M*_, and each resistance allele is gained (lost) at rate *ν*_*R*_ (*μ*_*R*_) independently. The fitness of genotypes is *w* = 1 under no stress. In the presence of a stress, the corresponding resistance mutation provides a selective advantage *s* > 0. If multiple stresses occur simultaneously (as is the case in Fig 3B), the fitness advantages due to resistance to the individual stresses are assumed to be additive. There are no cross-resistances, i.e., each resistance allele confers resistance against exactly one stress.

## Supporting information

S1 Appendix Modelling and simulation details. This text provides additional information about the model, a derivation of the analytic results in Eq (4), and details about simulations of an additional parameter set.
(PDF)

S2 Appendix Extensions of the model. In this text, we discuss how a cost of resistance and the possibility of lethal mutations impact our model.
(PDF)

S3 Appendix Heuristic prediction for multiple stresses. Based on the intuitive derivation of the dynamics of SIM allele frequency *p*_*M*_ in the main text, we present a heuristic prediction for the long-term SIM allele frequencies with *χ* > 1 stresses and compare it to numerical simulations.
(PDF)

S4 Appendix Resistance frequencies for different combination strategies. We show how different combination strategies affect the fraction of individuals that are multi-resistant.
(PDF)
