# Supplementary material for: Stress-induced mutagenesis: Stress diversity facilitates the persistence of mutator genes
Source: PLoS Comput Biol. 2017 Jul 18;13(7):e1005609. doi: 10.1371/journal.pcbi.1005609 (PMC5538753; doi:10.1371/journal.pcbi.1005609)
Supplement: S1 Appendix — This text provides additional information about the model, a derivation of the analytic results in Eq (4), and details about simulations of an additional parameter set. (PDF) [file pcbi.1005609.s001.pdf]

S1 Appendix. Modelling and simulation details

Analytical treatment

The no-stress environment. In terms of the frequency of the SIM allele, $p_M = p_{Mr} + p_{MR}$, the frequency of resistant genotypes, $p_R = p_{mR} + p_{MR}$, and the frequency of resistant genotypes among those that carry the SIM allele, $q = p_{MR}/p_M$, we may rewrite Eq (7) as

$$\begin{cases} \dot{p}_M &= -\mu_M\, p_M \\ \dot{p}_R &= \nu_R\,(1-p_R) - \mu_R\, p_R \\ \dot{q} &= \nu_R\,(1-q) - \mu_R\, q \end{cases} . \tag{A}$$

Remarkably, these equations are independent of each other. Furthermore, the equations for p_R and q are identical; given the same initial conditions $p_R(0)$ and $q(0)$, these two variables thus remain the same. The solution to Eq (A) is

$$\begin{cases} p_M(t) &= p_M(0)\, e^{-\mu_M t} \\ p_R(t) &= \left(1 - e^{-(\mu_R+\nu_R)t}\right) \frac{\nu_R}{\mu_R+\nu_R} + p_R(0)\, e^{-(\mu_R+\nu_R)t} \\ q(t) &= \left(1 - e^{-(\mu_R+\nu_R)t}\right) \frac{\nu_R}{\mu_R+\nu_R} + q(0)\, e^{-(\mu_R+\nu_R)t} \end{cases} . \tag{B}$$

The stress environment. To obtain tractable equations for the stress environment, we assume that s and σ are large, and that the duration of stress is short relative to the duration of no stress. As indicated in the Methods section, we take the full set of equations, Eq (8), replace $s \mapsto \alpha s$, $\sigma \mapsto \alpha\sigma$, and rescale time $dt \mapsto dt/\alpha$. Then, dividing by α and letting $\alpha \to \infty$, we may neglect the rightmost terms to obtain

$$\begin{cases} \dot{p}_{mr} &= -s\, p_{mr}\,(p_{mR} + p_{MR}) + \sigma\mu_M\, p_{Mr} \\ \dot{p}_{Mr} &= -s\, p_{Mr}\,(p_{mR} + p_{MR}) - \sigma\,(\mu_M + \nu_R)\, p_{Mr} \\ \dot{p}_{mR} &= s\, p_{mR}\,(1 - p_{mR} - p_{MR}) \\ \dot{p}_{MR} &= s\, p_{MR}\,(1 - p_{mR} - p_{MR}) + \sigma\nu_R\, p_{Mr} \end{cases} . \tag{C}$$

This approximation corresponds to neglecting all mutational transitions that are not multiplied by σ in Fig 1B. Evidently, this dynamics converges to a unique equilibrium where all genotypes are resistant, and some fraction of genotypes containing the SIM allele. In terms of the variables introduced above, this corresponds to $p_R(t) \to 1$, $q(t) \to 1$, and $p_M(t) \to p_M^*$ for $t \to \infty$.

To calculate an expression for the SIM allele frequency after stress p_M^* analytically, we recast the system Eq (C) using the variables $p_R = p_{mR} + p_{MR}$, $y = p_{Mr}/p_R$, and $z = p_{mR}/p_R$:

$$\begin{cases} \dot{p}_R &= s\, p_R\,(1 - p_R) + \sigma\,\nu_R\, p_R\, y \\ \dot{y} &= -y\,[s + \sigma\,(\mu_M + \nu_R\,(1+y))] \\ \dot{z} &= -\sigma\nu_R\, y\, z \end{cases} . \tag{D}$$

For given initial conditions $(p_R(0), y(0), z(0))$ and $t \to \infty$, this system converges to $p_R(t) \to 1$, $y(t) \to 0$, and

$$z(t) \to z_\infty = z(0)\, \frac{s + \sigma\,(\mu_M + \nu_R)}{s + \sigma\,(\mu_M + \nu_R\,(1 + y(0)))}. \tag{E}$$

The expression for p_M^* is then given by $p_M^* = (1 - z_\infty)$.

Recursions for the SIM allele frequency. In both the (R) and (NR) regime, we measure the genotype frequencies directly before each stress to obtain the SIM allele frequency p_M' after one cycle of stress and no stress by

$$p_M' = (\mathcal{G} \circ \mathcal{F})\,(p_M), \tag{F}$$

where $\mathcal{F}$ and $\mathcal{G}$ are two mappings that describe the stress and no-stress phases, respectively. Throughout, we use the approximation of the stress dynamics from the previous paragraph, describing it by an instantaneous jump in the SIM allele frequency, $p_M \to \mathcal{F}(p_M) = p_M^*$. Thus, selection is assumed to be strong enough to fix the resistance allele practically immediately. Furthermore, if the stress does not persist for long, mutations from p_{MR} to p_{mR} can be neglected, and $p_M^* = (1 - z_\infty)$ (with z_∞ from Eq (E)) can be expected to approximate the full dynamics Eq (8) (Fig 1B) well.

We assume that one iteration of stress and no stress takes $\tau = \tau_S + \tau_{NS}$ time units. In our analytical approach here, stress is approximated by an instantaneous jump in allele frequencies, hence $\tau_S = 0$, and we apply the no-stress environment for $\tau_{NS} = \tau$ time units. Thus, the mapping $\mathcal{G} = \mathcal{G}_\tau$ depends explicitly on τ; due to equation (B), we have

$$\mathcal{G}(P) = \mathcal{G}_\tau(P) = P\, e^{-\mu_M \tau}. \tag{G}$$

The mappings of the jumps for the two stress regimes, $\mathcal{F}^{(R)}$ and $\mathcal{F}^{(NR)}$, will be defined below.

Note that, in contrast to the approximation described here, numerical simulations of the full dynamics, i.e., iterating Eq (8) for the stress environment and Eq (7) for the no-stress environment, naturally require $\tau_S > 0$ and $\tau_{NS} = \tau - \tau_S$. However, a comparison between our analytical results and simulations of the full dynamics for identical values of τ demonstrates a good fit between the two approaches, indicating that the approximations made here are justified (see Fig 2).

The recurrent stress regime. Suppose that the same stress occurs every $\tau > 0$ time units. Since we assume that each stress phase leads to the fixation of the resistance allele, we have that $p_R = q = 1$ at the beginning of each no-stress phase. Hence, because the equations of these two variables are identical, see Eq (A), we have $p_R(t) = q(t)$ for all times after the first occurrence of stress. At the end of each no-stress period, we thus have

$$p_R(\tau) = q(\tau) = \frac{\nu_R}{\mu_R + \nu_R} + e^{-(\mu_R+\nu_R)\tau}\left(1 - \frac{\nu_R}{\mu_R + \nu_R}\right) \tag{H}$$

due to Eq (B). Inserting these values to obtain new initial frequencies for the next stress phase required for Eq (E) allows us to calculate z_∞ and thus

$$\begin{aligned} \mathcal{F}^{(R)}(P) &= 1 - z_\infty = \\ &= 1 - \frac{(p_R(\tau) - P\,q(\tau))\,(s + \sigma\,(\mu_M + \nu_R))}{s\,p_R(\tau) + \sigma\,[p_R(\tau)\,(\mu_M + \nu_R) + \nu_R\,P\,(1 - q(\tau))]}. \end{aligned} \tag{I}$$

Inserting this expression and the identity Eq (G) into the general recursion Eq (F) yields Eq (3). Using the identity Eq (H) and solving for equilibria fulfilling $\hat{p}_M^{(R)} = \left(\mathcal{G} \circ \mathcal{F}^{(R)}\right)\left(\hat{p}_M^{(R)}\right)$ provides the long-term prevalence of the SIM allele in the recurrent stress regime, $\hat{p}_M^{(R)}$, as given in Eq (4a).

The non-recurrent stress regime. Suppose that the population never experiences the same stress twice. As a consequence, we may neglect any resistance gained from previous stress occurrences. Instead, we assume that the fraction of genotypes that initially are resistant against an upcoming stress is in mutation balance, i.e., determined by the relative rates of gaining and losing resistance by mutation. Hence, we may use $p_R = q = \nu_R/\left(\mu_R + \nu_R\right)$ instead of Eq (H) to calculate an expression for $\mathcal{F}^{(NR)}$ analogously to the above. Solving $\hat{p}_M^{(NR)} = \left(\mathcal{G} \circ \mathcal{F}^{(NR)}\right)\left(\hat{p}_M^{(NR)}\right)$, we obtain the long-term prevalence of the SIM allele in the non-recurrent stress regime, $\hat{p}_M^{(NR)}$, as given in Eq (4b).

Comparison between stress regimes. We assign the following names to the non-trivial terms on the right hand sides of Eq (4):

$$\mathcal{P}_\tau^{(R)} = e^{-\mu_M \tau} - \Gamma\left(1 - e^{-\mu_M \tau}\right)\left(1 + \frac{\mu_R + \nu_R}{\nu_R}\left(e^{(\mu_R+\nu_R)\tau} - 1\right)^{-1}\right), \tag{Ja}$$

$$\mathcal{P}_\tau^{(NR)} = e^{-\mu_M \tau} - \Gamma\left(1 - e^{-\mu_M \tau}\right), \tag{Jb}$$

where $\Gamma > 0$ is defined in Eq (5). Since

$$\Delta = \mathcal{P}_\tau^{(NR)} - \mathcal{P}_\tau^{(R)} = \frac{1 - e^{-\mu_M \tau}}{e^{(\mu_R+\nu_R)\tau} - 1}\,\frac{\mu_R + \nu_R}{\nu_R}\,\Gamma > 0, \tag{K}$$

the long-term SIM allele prevalence under non-recurrent stresses is never lower than in the recurrent stress regime ($\hat{p}_M^{(NR)} \geq \hat{p}_M^{(R)}$). In particular, for $\tau = \tau_c$ the value of Δ, and hence of $\mathcal{P}_{\tau_c}^{(R)}$, is already negative.

The SIM allele cannot be maintained in the population in the recurrent stress regime if ν_R is sufficiently small compared to μ_R. To see this, we rewrite Eq (K) as

$$\Delta = \frac{1}{\varepsilon}\,\frac{1 - e^{-\mu_M \tau}}{e^{(\mu_R+\nu_R)\tau} - 1}\,\Gamma. \tag{L}$$

Then, $\nu_R \ll \mu_R$ corresponds to $\varepsilon \ll 1$. Furthermore, on the closed interval $[0, \tau_c]$, the function

$$\frac{1 - e^{-\mu_M \tau}}{e^{(\mu_R+\nu_R)\tau} - 1}\,\Gamma$$

is bounded away from zero, i.e., it has a positive minimum. Therefore, if ν_R is small, $1/\varepsilon$ is large, and hence Δ is large on $[0, \tau_c]$. Thus, by choosing ν_R sufficiently small, we may push $\mathcal{P}_\tau^{(R)}$ arbitrarily far below zero, thus $\hat{p}_M^{(R)} \equiv 0$ for all $\tau \geq 0$.

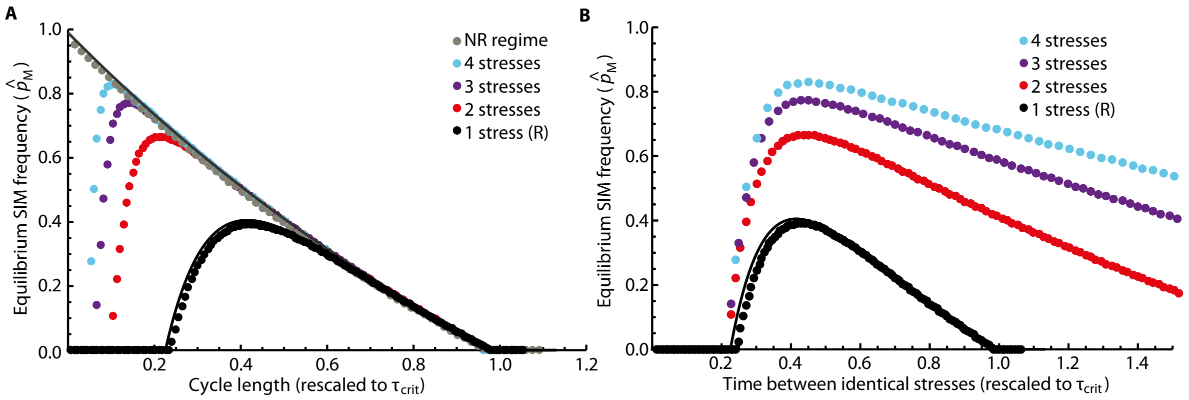

Figure A: Long-term prevalence of the SIM allele for parameters conflicting the ranking (2), $\sigma = 100$, $s = 1$, $\mu_M = 5 \times 10^{-4}$, $\mu_R = 10^{-2}$, and $\nu_R = 10^{-4}$. We chose $\tau_S = 10$ and varied τ_{NS} accordingly. Unlike in Figure 2, the SIM allele is maintained for a certain range of values of τ. The black and grey points are the simulation results for the (R) and (NR) regimes; they closely match their analytical predictions from equation (4), represented by solid black lines. As expected, an increasing number of stresses increases the long-term SIM allele prevalences and the interval of τ under which the SIM allele is maintained.

Simulations of an additional parameter set

Under our assumptions in Eq (2), we showed that the SIM allele is always lost under the recurrent stress (R) regime. A numerical example with $\sigma = 100$, $s = 1$, $\mu_M = 10^{-3}$, $\mu_R = 10^{-2}$, and $\nu_R = 10^{-4}$ is shown in Fig 2 in the main text. If the SIM allele decays only at half the rate relative to this example, $\mu_M = 5 \times 10^{-4}$, the basic ranking of parameters is violated, since we now have $\mu_M \approx \nu_R$. Fig A shows that in this case the SIM allele is maintained for an interval of stress re-occurrence times τ. The fit with our analytical predictions, Eq (4), remains very good and the qualitative patterns with increasing stress diversity is unchanged.
