# Supplementary material for: Stress-induced mutagenesis: Stress diversity facilitates the persistence of mutator genes
Source: PLoS Comput Biol. 2017 Jul 18;13(7):e1005609. doi: 10.1371/journal.pcbi.1005609 (PMC5538753; doi:10.1371/journal.pcbi.1005609)
Supplement: S2 Appendix — In this text, we discuss how a cost of resistance and the possibility of lethal mutations impact our model. (PDF) [file pcbi.1005609.s002.pdf]

S2 Appendix. Extensions of the model

We may include additional biological features into our model by considering, e.g., a cost of resistance in the absence of stress, or the occurrence of lethal deleterious mutations that may be amplified by the SIM allele. These modifications change the SIM allele prevalences in intuitive ways, yet do not interfere with the qualitative results that are in the focus of the main text.

Cost of resistance

If resistance is costly, the fitness of the mR and MR genotypes is reduced in the absence of stress. All else staying equal, reduce the fitness of those genotypes under no stress from $w = 1$ to $w = 1 - c$. As a consequence, the dynamics in Eq (A) change to

$$\left\{ \begin{array}{rcl} \dot{p}_M &=& c p_M \left(p_R - q\right) - \mu_M \, p_M \\ \dot{p}_R &=& -c \, p_R \left(1 - p_R\right) + \nu_R \left(1 - p_R\right) - \mu_R \, p_R \\ \dot{q} &=& -c \, q \left(1 - q\right) \nu_R \left(1 - q\right) - \mu_R \, q \end{array} \right. . \tag{A}$$

In the (NR) regime, a cost of resistance does not change the results, since the resistance levels to previous stresses are irrelevant, and selection against resistant genotypes does not change the proportions of the two susceptible genotypes mr and Mr relative to each other.

For the (R) regime, we need to only slightly modify our previous analysis. Analogously to our reasoning above, $p_R(t) = q_R(t)$ after the first occurrence of stress. Consequently, the dynamics of p_M simplifies to $\dot{p}_M = -\mu_M \, p_M$, thus the mapping $\mathcal{G}$ in Eq (G) does not change. Since the dynamics for the stress period are unchanged, also the map $\mathcal{F}^{(R)}$, Eq (I), remains the same. An expression for $p_R(\tau) = q(\tau)$ (c.f. Eq (H)) can be calculated explicitly and solving $\hat{p}_M^{(R)} = \left(\mathcal{G} \circ \mathcal{F}^{(R)}\right) (\hat{p}_M^{(R)})$ for $\hat{p}_M^{(R)}$ yields the long-term prevalence of the SIM allele under recurrent stress and with a cost c of resistance. The exact expressions are complicated, but numerical simulations show that a cost of resistance increases the long-term prevalence of the SIM allele, see Fig B. This is intuitively clear: a cost to resistance augments the decay of resistance levels in the absence of stress, hence increases the benefit of increased mutation rates to acquire resistance *de novo*.

For weak cost of resistance c, we may do a first-order approximation of the long-term SIM prevalence to obtain

$$\hat{p}_M^{(R)} = \max \left\{ 0 \, , \, \mathcal{P}_\tau^{(R)} + c \, \Gamma \, \frac{e^{-\mu_M \tau} (e^{\mu_M \tau} - 1)}{\left(1 - e^{(\mu_R + \nu_R)\tau}\right)^2 (\mu_R + \nu_R) \, \nu_R} \, \xi + \mathcal{O}(c^2) \right\} \tag{B}$$

(c.f. Eq (Ja)), where $\xi = \mu_R + \nu_R e^{2(\mu_R + \nu_R)\tau} + e^{(\mu_R + \nu_R)\tau} \left((\mu_R - \nu_R)\tau - 1\right) (\mu_R + \nu_R)$ can be shown to be positive for $\tau > 0$. Hence, the long-term prevalence levels of the SIM allele under a weak cost c of resistance increase with c.

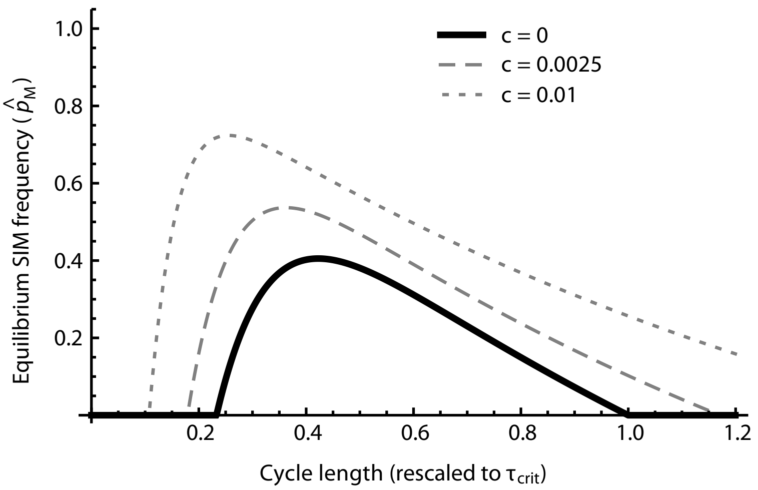

Figure B: Long-term prevalence of the SIM allele with a cost of resistance. For the parameters of Fig A, the solid black line shows the long-term SIM prevalence in the (R) regime as a function of cycle length τ without a cost of resistance ($c = 0$). A cost of resistance causes resistance levels in the no-stress phase to decay more rapidly, which increases the benefit provided by the SIM allele. Consequently, we observe elevated equilibrium SIM frequencies (dashed and dotted lines for $c = 0.0025$ and $c = 0.01$, respectively).

Lethal mutations

High mutation rates potentially lead to a substantial mutation load due to deleterious mutations. Modelling the gradual accumulation of deleterious mutations requires multiple fitness classes, which is infeasible in our model. However, we may consider the extreme case of lethal mutations and investigate their impact on the dynamics of the SIM allele.

Assume that lethal mutations occur at rate δ, and that this rate is amplified to $\sigma\delta$ in Mr genotypes under stress. In the absence of stress, this does not change the dynamics of genotype frequencies, because all genotypes incur lethal mutations at the same rate, which keeps their relative frequencies intact. Under stress, the abundances $\{n_{mr}, n_{Mr}, n_{mR}, n_{MR}\}$ evolve according to

$$\dot{n}_{mr} = -\nu_R\, n_{mr} + \mu_R\, n_{mR} + \sigma\mu_M\, n_{Mr} - \delta\, n_{mr}, \tag{Ca}$$

$$\dot{n}_{Mr} = -\sigma(\mu_R + \nu_R)\, n_{Mr} + \mu_R\, n_{MR} - \sigma\delta\, n_{Mr}, \tag{Cb}$$

$$\dot{n}_{mR} = -\mu_R\, n_{mR} + \nu_R\, n_{mr} + \mu_M\, n_{MR} - \delta\, n_{mR}, \tag{Cc}$$

$$\dot{n}_{MR} = -(\mu_M + \mu_R)\, n_{MR} + \sigma\nu_R\, n_{Mr} - \delta\, n_{MR}, \tag{Cd}$$

which translates into genotype frequencies as

$$\dot{p}_{mr} = -s\, p_{mr}\,(p_{mR} + p_{MR}) + \sigma\mu_M\, p_{Mr} - \nu_R\, p_{mr} + \mu_R\, p_{mR} + \boldsymbol{\delta\,(\sigma - 1)\, p_{mr}\, p_{Mr}}, \tag{Da}$$

$$\dot{p}_{Mr} = -s\, p_{Mr}\,(p_{mR} + p_{MR}) - \sigma\,(\mu_M + \nu_R)\, p_{Mr} + \mu_R\, p_{MR} - \boldsymbol{\delta\,(\sigma - 1)\, p_{Mr}\,(1 - p_{Mr})}, \tag{Db}$$

$$\dot{p}_{mR} = s\, p_{mR}\,(1 - p_{mR} - p_{MR}) + \nu_R\, p_{mr} - \mu_R\, p_{mR} + \mu_M\, p_{MR} + \boldsymbol{\delta\,(\sigma - 1)\, p_{mR}\, p_{Mr}}, \tag{Dc}$$

$$\dot{p}_{MR} = s\, p_{MR}\,(1 - p_{mR} - p_{MR}) + \sigma\nu_R\, p_{Mr} - (\mu_R + \mu_M)\, p_{MR} + \boldsymbol{\delta\,(\sigma - 1)\, p_{MR}\, p_{Mr}} \tag{Dd}$$

(c.f. Eq (8) in the main text). Hence, lethal mutations take the form of additional selection terms (highlighted in bold) against the Mr genotype.

We set $\beta = \sigma\delta$ and assume that s and σ are large, and that the duration of stress is short relative to the duration of no stress. Analogously to the above treatment without lethal mutations, we then obtain

$$\begin{cases} \dot{p}_R &= s\, p_R\,(1 - p_R) + \sigma\,\nu_R\, p_R\, y + \beta\, p_R^2\, y \\ \dot{y} &= -y\,[s + \beta + \sigma\,(\mu_M + \nu_R\,(1 + y))] \\ \dot{z} &= -\sigma\nu_R\, y\, z \end{cases}. \tag{E}$$

Similarly to Eq (D), this system can be solved for given initial conditions $(p_R(0), y(0), z(0))$. In the limit of $t \to \infty$, we have $p_R(t) \to 1$, $y(t) \to 0$, and

$$z(t) \to z_\infty = z(0)\,\frac{s + \beta + \sigma\,(\mu_M + \nu_R)}{s + \beta + \sigma\,(\mu_M + \nu_R\,(1 + y(0)))}. \tag{F}$$

Comparing to Eq (E), we see that lethal mutations are incorporated into the model simply by replacing $s \mapsto s + \beta$. Consequently, lethal mutations increase the parameter Γ, c.f. Eq (5), and hence the long-term prevalence of the SIM allele as expected. This is illustrated in Fig C for the parameters used in Fig A and different values of β.

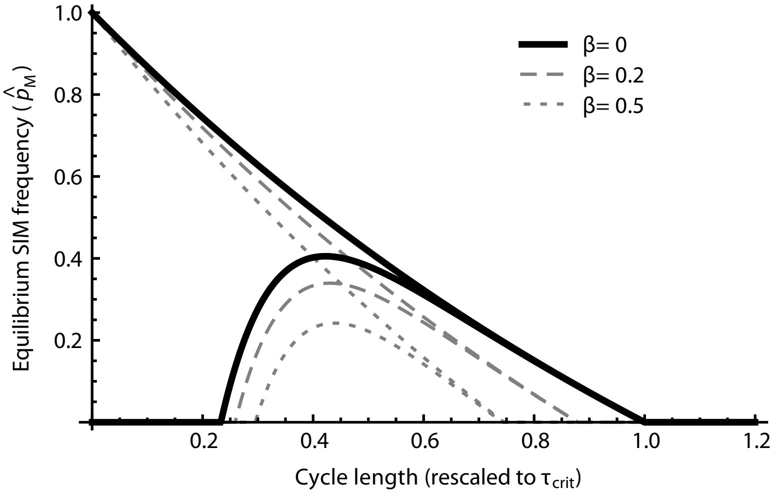

Figure C: Long-term prevalence of the SIM allele with lethal mutations. For the parameters of Fig A, the solid black lines show the long-term SIM prevalences in the (R) and (NR) regimes as functions of cycle length τ in the absence of lethal mutations ($\beta = \sigma\delta = 0$). Increased mutation rates during stress also increase the deleterious mutation load for the Mr genotype, hence reducing the equilibrium frequency of the SIM allele (dashed and dotted lines for $\beta = 0.2$ and $\beta = 0.5$, respectively).
