# Supplementary material for: Stress-induced mutagenesis: Stress diversity facilitates the persistence of mutator genes
Source: PLoS Comput Biol. 2017 Jul 18;13(7):e1005609. doi: 10.1371/journal.pcbi.1005609 (PMC5538753; doi:10.1371/journal.pcbi.1005609)
Supplement: S3 Appendix — Based on the intuitive derivation of the dynamics of SIM allele frequency pM in the main text, we present a heuristic prediction for the long-term SIM allele frequencies with χ > 1 stresses and compare it to numerical simulations. (PDF) [file pcbi.1005609.s003.pdf]

S3 Appendix. Heuristic prediction for multiple stresses

For $1 < \chi < \infty$ different stresses, the number of genotypes to consider is $2^{\chi+1}$. Already for $\chi = 2$, the exact dynamics of genotype frequencies becomes intractable, even under our simplifying assumptions. However, we may modify the intuitive derivation of the central recursion, Eq (3), presented in the main text to approximate the dynamics of SIM allele frequencies with $\chi > 1$ stresses. When stresses re-occur cyclically, the time between two stresses of the same kind is $\chi\tau$. If there were no interactions between resistance alleles against different stresses (e.g. due to cross-resistant genotypes against multiple stresses), the frequency of resistant genotypes against any specific stress before its re-occurrence would be given by $p_R(\chi\tau)$, where p_R is given above in Eq (H). Thus, replacing $p_R(\tau_{NS})$ by $p_R(\chi\tau_{NS})$ in the recursion Eq (3) and solving for p_M yields an approximation to the long-term SIM allele frequency in a model with χ different stresses. It correctly captures the qualitative behaviour of the system, yet overestimates the long-term SIM allele frequencies obtained from numerical simulations, see Fig D.

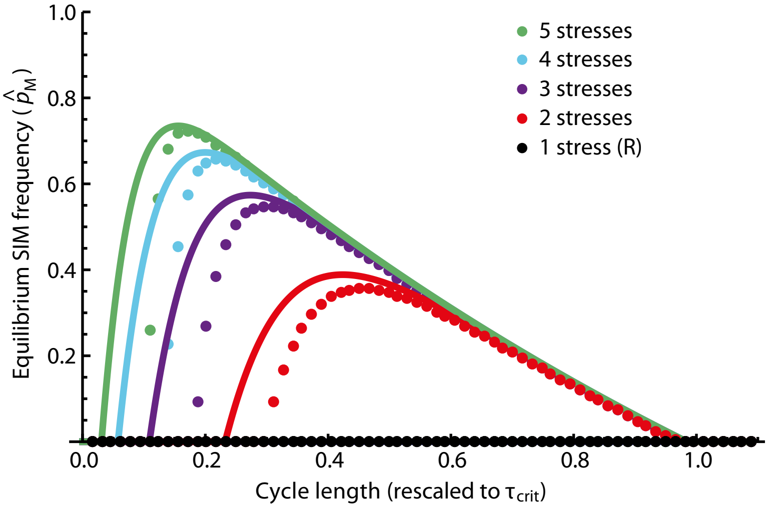

Figure D: Long-term prevalence of the SIM allele with multiple stresses. This figure shows the simulated long-term SIM allele frequencies (coloured points) for the parameters used in Fig 2 for $\chi = 1, ..., 5$ stresses. If $\chi = 1$ (the recurrent (R) regime), the SIM allele is not maintained. For $\chi > 1$, the long-term SIM allele frequencies increase as described in the main text. The heuristic prediction described in S3 Appendix is represented by solid lines. While the approximation is good for $\tau \approx \tau_c$, it overestimates the simulated long-term SIM allele frequencies for shorter cycle lengths τ.
