# Supplementary material for: Stress-induced mutagenesis: Stress diversity facilitates the persistence of mutator genes
Source: PLoS Comput Biol. 2017 Jul 18;13(7):e1005609. doi: 10.1371/journal.pcbi.1005609 (PMC5538753; doi:10.1371/journal.pcbi.1005609)
Supplement: S4 Appendix — We show how different combination strategies affect the fraction of individuals that are multi-resistant. (PDF) [file pcbi.1005609.s004.pdf]

S4 Appendix. Resistance frequencies for different combination strategies

Here we investigate the effectiveness of different combination treatments at avoiding the evolution to multiple resistances. We use the same parameters as in the main text and show how simultaneously presenting a different number of stresses impacts on the frequency of multi-resistant strains. We calculate these frequencies for three different cycle lengths ($\tau_{NS} = 600, 1200$ and 2400) corresponding to the regimes in which the SIM allele is not maintained for none of the treatments ($t = 1$ in Fig 3B), the SIM allele is maintained for split treatments ($t = 2$ in Fig 3B) and to the regime where the cycle length is too long to maintain the SIM allele ($t = 4$ in Fig 3B).

We find that presenting all stresses simultaneously is in general more effective at maintaining low levels of resistance, but can lead to high levels of multi-resistance if the non-stress period (τ_{NS}) is short. Intercalating different subsets of stresses seems to be the best compromise at avoiding the rise of multi-resistant genotypes.

Pattern	Single stress only	Exactly 2 stresses	All 4 stresses
$\tau_{NS} = 600$		(no SIM allele maintained)	
(1,2,3,4)	0.212717	0.212717	0.212717
(1,2)–(3,4)	0.379765	0.0889999	0.000193944
(1)–(2)–(3)–(4)	0.471306	0.154693	0.000518945
$\tau_{NS} = 1200$		(Split strategies maintain SIM allele)	
(1,2,3,4)	0.0813984	0.00271294	$2.23266 \cdot 10^{-7}$
(1,2)–(3,4)	0.153122	0.00955676	$1.94671 \cdot 10^{-6}$
(1)–(2)–(3)–(4)	0.281112	0.0246651	$7.12363 \cdot 10^{-6}$
$\tau_{NS} = 2400$		(no SIM allele maintained)	
(1,2,3,4)	0.0739242	0.00221827	$1.47949 \cdot 10^{-7}$
(1,2)–(3,4)	0.0776765	0.00245756	$1.81719 \cdot 10^{-7}$
(1)–(2)–(3)–(4)	0.115715	0.00492387	$5.4031 \cdot 10^{-7}$

Table A: Fraction of the population that is resistant to 1,2 or 4 stresses for different combination treatments and intervals between stress periods (τ_{NS}). (1,2,3,4) means that all stresses are applied simultaneously; (1,2)–(3,4) means that stresses 1 and 2 are applied simultaneously, and after a period of τ_{NS}, stresses 3 and 4 are applied; (1)–(2)–(3)–(4) means all stresses are applied independently. $\tau_{NS} = 600$ corresponds to time point 1 in figure 3B, $\tau_{NS} = 1200$ to time point 2 and $\tau_{NS} = 2400$ to time point 4. Resistance levels are measured at the beginning of each stress phase (and are the same for every phase, so only one number is reported).
